# Soil microorganisms and methane emissions in response to short-term warming field incubation in Svalbard

**DOI:** 10.3389/fmicb.2023.1276065

**Published:** 2023-11-24

**Authors:** Jiakang Li, Zhuo-Yi Zhu, Zhifeng Yang, Weiyi Li, Yongxin Lv, Yu Zhang

**Affiliations:** ^1^Shanghai Key Laboratory of Polar Life and Environment Sciences, School of Oceanography, Shanghai Jiao Tong University, Shanghai, China; ^2^School of Environmental Science and Engineering, Shanghai Jiao Tong University, Shanghai, China; ^3^MNR Key Laboratory for Polar Science, Polar Research Institute of China, Shanghai, China; ^4^Department of Microbiology and Plant Biology, University of Oklahoma, Norman, OK, United States; ^5^School of Life Sciences and Biotechnology, Shanghai Jiao Tong University, Shanghai, China

**Keywords:** Arctic soil, warming, field incubation, microorganisms, methane

## Abstract

**Introduction:**

Global warming is caused by greenhouse gases (GHGs). It has been found that the release of methane (CH_4_) from Arctic permafrost, soil, ocean, and sediment is closely related to microbial composition and soil factors resulting from warming over several months or years. However, it is unclear for how long continuous warming due to global warming affects the microbial composition and GHG release from soils along Arctic glacial meltwater rivers.

**Methods:**

In this study, the soil upstream of the glacial meltwater river (GR) and the estuary (GR-0) in Svalbard, with strong soil heterogeneity, was subjected to short-term field incubation at 2°C (*in situ* temperature), 10°C, and 20°C. The incubation was carried out under anoxic conditions and lasted for few days. Bacterial composition and CH_4_ production potential were determined based on high-throughput sequencing and physiochemical property measurements.

**Results:**

Our results showed no significant differences in bacterial 16S rRNA gene copy number, bacterial composition, and methanogenic potential, as measured by mcrA gene copy number and CH4 concentration, during a 7- and 13-day warming field incubation with increasing temperatures, respectively. The CH_4_ concentration at the GR site was higher than that at the GR-0 site, while the *mcrA* gene was lower at the GR site than that at the GR-0 site.

**Discussion:**

Based on the warming field incubation, our results indicate that short-term warming, which is measured in days, affects soil microbial composition and CH_4_ concentration less than the spatial scale, highlighting the importance of warming time in influencing CH_4_ release from soil. In summary, our research implied that microbial composition and CH_4_ emissions in soil warming do not increase in the first several days, but site specificity is more important. However, emissions will gradually increase first and then decrease as warming time increases over the long term. These results are important for understanding and exploring the GHG emission fluxes of high-latitude ecosystems under global warming.

## 1 Introduction

Arctic terrestrial ecosystems currently store the largest amounts of carbon in the high-latitude regions of the Earth. Over the last 30 years, the temperature levels of these regions have risen twice as fast as the global average, at 0.6°C per decade (Cohen et al., 2014; Schuur et al., 2015). It is a robust phenomenon known as Arctic amplification (Fengmin et al., 2019). The soil microorganisms play an important role in converting carbon compounds into organic or inorganic compounds, and their metabolic rate increases due to warming. When microbes break down organic carbon, they release greenhouse gases (GHGs) such as carbon dioxide (CO_2_), nitrous oxide (N_2_O), and methane (CH_4_), leading to global climate change (Mehmood et al., 2020; Marushchak et al., 2021). In the past 800,000 years, the levels of atmospheric CO2, N2O, and CH4 have increased significantly. The current levels of these gases are 390.5 parts per million (ppm) for CO_2_, 390.5 parts per billion (ppb) for N_2_O, and 1,803.2 ppb for CH_4_, and these levels are, respectively, 40, 20, and 150% higher than they were before the industrial era (Tian et al., 2016; Mehmood et al., 2020). CH_4_, the second most important GHG after CO_2_, accounts for at least 20% of the anthropogenic radiative forcing of warming agents since the preindustrial era. Moreover, the greenhouse effect of CH_4_ is 28 times that of CO_2_ in 100 years (Tian et al., 2016; Ganesan et al., 2019; Hui et al., 2020). In the Arctic region, CH_4_ emissions range from 15 to 50 Tg/yr, as estimated by biogeochemistry models and atmospheric inversions between 2000 and 2017 (Saunois et al., 2016, 2020). Due to Arctic amplification, global climate change will lead to Arctic soil warming and CH_4_ emissions. However, the duration of the impact of warming on the CH_4_ release from the soil, causing climate change, is yet undiscovered.

Microbial metabolic processes have long been the key drivers and responders to climate change (Singh et al., 2010). According to research findings, different soil microorganisms produce GHGs through different metabolic pathways related to microbial composition, providing an improved understanding of GHG emissions. For example, most soil microorganisms contribute greatly to CO_2_ emissions through decomposition and heterotrophic respiration (Watts et al., 2021). Similar to CO_2_ emissions, biotic CH_4_ emissions are controlled by soil microbial methanogenesis and CH_4_ oxidation from the soil, lake, and other terrestrial places, especially Arctic soil (Nazaries et al., 2013; Tveit et al., 2013; Hamdan and Wickland, 2016; Knoblauch et al., 2018; Galera et al., 2023). Microbial methanogenesis is a process carried out by a group of anaerobic methanogenic archaea (Song et al., 2021). While the other microorganisms can catabolize CH_4_, thus easing the release of CH_4_ into the atmosphere, microbial methanogenesis contributes greatly to global CH_4_ emissions, and understanding its response to warming time is fundamental to predicting the feedback between potent GHGs and climate change (Lee et al., 2012; Chen et al., 2020). Moreover, the microbial composition was expected to change under long-term warming measured by years (Deslippe et al., 2012; Pold et al., 2021; Zosso et al., 2021; Rijkers et al., 2022; Zhou et al., 2023). Meanwhile, biotic CH_4_ emissions are also caused by warming through long-term microbial fermentation (Altshuler et al., 2019; Hui et al., 2020; Zhang et al., 2021). However, climate change is a process that accumulates over time; therefore, the duration of its impact on the environment is unknown. All global climate processes are based on and originate from short-term climate changes. Short-term processes start all long-term processes, and the feedback of short-term processes is more rapid and direct. Long-term processes are the net effect of accumulation and comprehensive influence of many short-term processes. Nevertheless, an analysis of microbial composition and CH_4_ emissions due to short-term warming, measured in days, will help us understand the effects of warming on releasing greenhouse gases from the soil.

In addition to temperature and microbial control, soil characteristics, such as moisture, oxygen concentration, and vegetation types (substrates), are recognized as important drivers of the CH_4_ emission fluxes (Nazaries et al., 2013; Voigt et al., 2019; Song et al., 2021). Warming can affect carbon emissions by altering the concentration of nutrients and the rate of decomposition of organic matter (Pareek, 2017). Simultaneously, soil moisture is closely related to the aerobic/anoxic boundary and may also vary with the evapotranspiration stimulated by warming, which eventually affects aerobic respiration and anaerobic methanogenesis (Zhang et al., 2023b). Consequently, wetter areas caused by future climate conditions will have higher moisture content, creating anaerobic conditions that increase CH_4_ production and, at the same time, reduce CH_4_ consumption by reducing O_2_ production (Singh et al., 2010; Lawrence et al., 2015). Treat et al. (2014) found that, in terms of active-layer thickness, CO_2_ and CH_4_ emissions from peat depth ranged from 77% greater than to not significantly different from permafrost depths. This variation depends on the peat type and peat decomposition stage rather than the thermal state, as determined through an incubation experiment. However, few studies have examined the impact of these environmental factors on GHG emissions, particularly CH_4_ emissions over warming periods in the Arctic.

Current studies about GHG emissions under Arctic soil warming focus on GHG release from soil affected by environmental factors (Elberling et al., 2008; Tian et al., 2016) and novel microbial communities (Wartiainen et al., 2003, 2006), which have been researched in the western Canadian Arctic (Barbier et al., 2012; Martineau et al., 2014). Few studies were carried out in Svalbard, except for the Ny-Alesund region (Tveit et al., 2015; Newsham et al., 2022). As mentioned previously, CH_4_ emissions are affected by vegetation type, soil substrates, and moisture. Correspondingly, the soil of the Svalbard Glacier basin has great heterogeneity (Son and Lee, 2022). For example, corresponding to the Ny-Alesund tundra landform, the Barentsburg region has higher vegetation coverage and longer glacial meltwater rivers (nearly 10 km) than the Ny-Alesund Bay River (about 3 km). Temperatures in Svalbard's topsoil can reach more than 10°C and even approach 20°C in summer (Cappelletti et al., 2022; Magnani et al., 2022). Therefore, short-term warming experiments can provide a foundation for studying the effects of warming on the microbial composition and GHG release from the soil in Barentsburg.

Based on the background, warming and anoxic field experiments in this study were carried out with the glacial meltwater river soil around Barentsburg for 7 and 13 days. The short-term warming was explored from the changes in the bacterial 16S rRNA gene copy number and composition, abundance of CH_4_-producing genes, and CH_4_ concentration in the soil, which affects bacterial diversity and CH_4_ emissions. From the perspective of incubation experiments, this study revealed the relationship between short-term warming and CH_4_ release from the soil near the upstream and estuary of the glacial meltwater river in Barentsburg.

## 2 Materials and methods

### 2.1 Soil samples and incubation experiments

**Figure 1:**
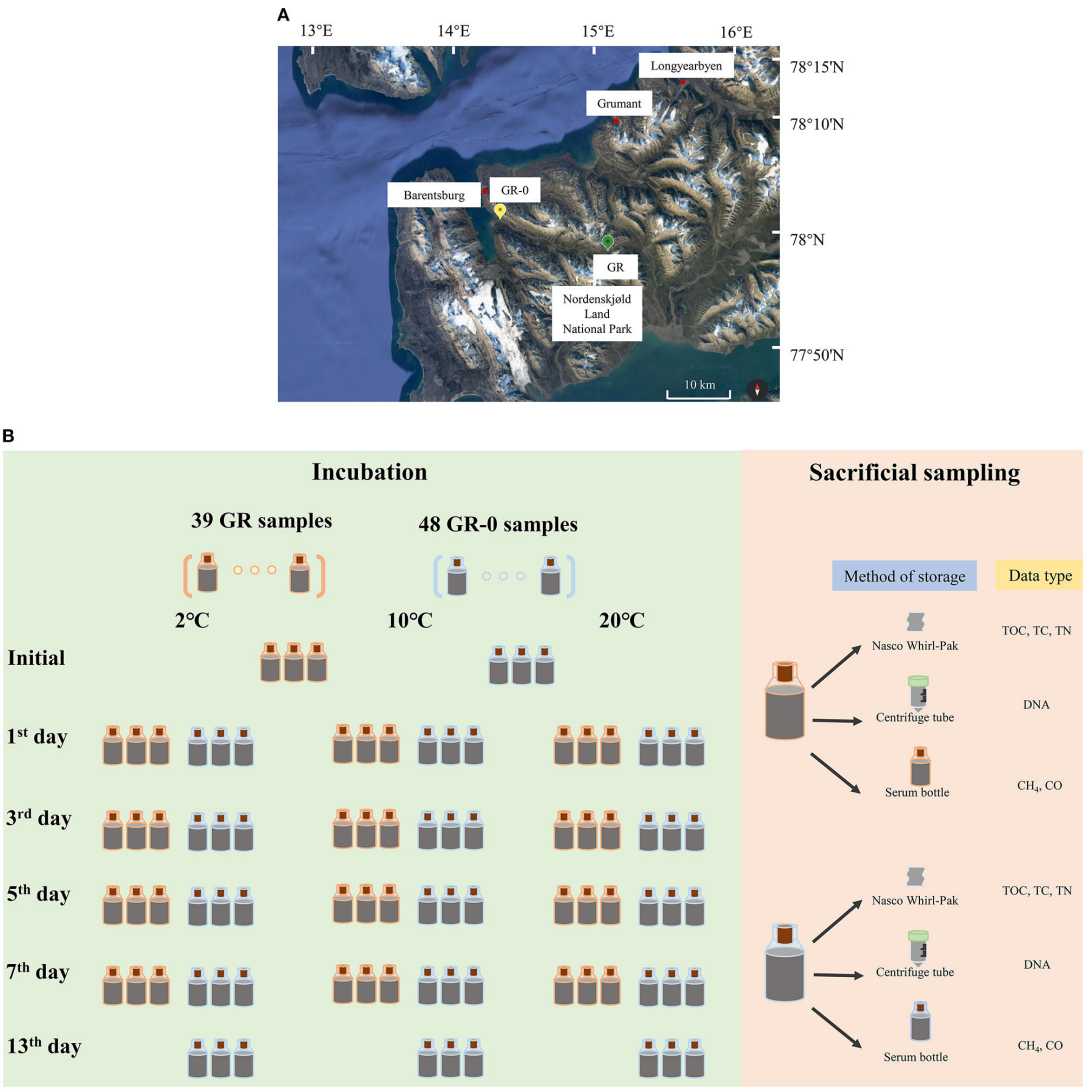
Sampling sites **(A)**, warming field incubation, and sampling process **(B)**.

We collected samples from the two sites near Barentsburg during July and August of 2018. One sample was collected from the soil (GR, 15°5′23.100“E, 77°58′39.173”N) upstream of the glacial meltwater river, while the other was taken from the soil (GR-0, 14°20′24.601“E, 78°1′29.143”N) at the estuary of the glacier meltwater river (Figure 1A) at 2°C. While in the field, we placed approximately 46 g of soil into 20 mL brown serum bottles (223762, Wheaton, USA) with a stopper and incubated them at three temperatures (2°C, 10°C, and 20°C). Bottles were filled to full, leaving no space with oxygen. After incubation, sacrificial sampling was taken on 0, 1, 3, 5, 7, and 13 days. The sample ID was named (GR/GR-0)-X-Y, where X represents the number of incubation days while Y represents the incubation temperature levels. Temperature readings were recorded at the *in situ* temperature of 2°C on days 1–3, 10°C on days 4–6, and 20°C on days 7–9. Approximately 8 g of the soil was stored in a Nasco Whirl-Pak sample bag (B01062WA, Nasco, USA) for measuring environmental parameters, and approximately 32 g of the soil was added into a 50 mL centrifuge tube with 20 mL RNA later (AM7021, Invitrogen, USA) for determining the composition of the microorganism community at 20°C. However, 3 mL of 2 mol/L NaOH was added to a 20-mL serum bottle along with 5 g soil for determining CH_4_ concentration and stored at 4°C (Figure 1B).

### 2.2 Bacterial community analyses

The genomic DNA of six initial samples and 81 incubation samples stored in RNAlater at −20°C was extracted from approximately 0.5 g fresh, homogenized soil using the FastDNA^®^ SPIN Kit for Soil (116560200, MP Biomedicals, USA). Before following the manufacturer's instructions, all the samples were washed with 1 × PBS twice and centrifuged at 12,000 × g for 5 min. The DNA concentration was then measured using a NanoDrop 2000 spectrometer (Thermo Fisher Scientific, USA). The V4 region of the bacterial 16S rRNA gene was amplified using the primers 533F (5′-TGCCAGCAGCCGCGGTAA-3′) and Bact 806R (5′-GGACTACHVGGGTWTCTAAT-3′) (Klindworth et al., 2012; Zhang et al., 2020) with an 8-bp unique barcode at the forward primer. The PCR procedure was performed in 50 μL reactions, which were repeated three times for increased accuracy. The thermal cycling conditions for the bacterial 16S *rRNA* gene involved an initial denaturation at 94°C for 5 min, followed by 25 cycles of denaturation at 94°C for 30 s, annealing at 58°C for 40 s, and extension at 72°C for 30 s with a final extension at 72°C for 10 min. The PCR products were gel-purified using an EZNA Gel Extraction Kit (Omega Bio-Tek, Inc., USA). The sequencing of purified DNA on the Illumina MiSeq platform was performed by the Personalbio Biotechnology company in Shanghai, China.

The analysis used the pipeline QIIME2 (version 2022.2) (Bolyen et al., 2019). First, the partial region was extracted using the corresponding primer set from the sequences in the SILVA (version 138) database and was used to train a classifier using the “feature-classifier” plugin. Then, all the datasets were grouped by the primer. After trimming of the corresponding primer, the sequencing quality of the raw reads was manually assessed to determine the appropriate truncated position for filtering low-quality regions. Paired-end reads were merged and dereplicated using the “dada2” plugin. Unassigned or eukaryotic ASVs were removed and the remaining ASVs were classified using the trained classifier.

### 2.3 Prepared templates for qPCR standard curves

The 16S *rRNA* gene copy number of bacteria was quantified by real-time quantitative polymerase chain reaction (qPCR) using bac341f (5′-CCTACGGGWGGCWGCA-3′) and prokaryotic 519r (5′-TTACCGCGGCKGCTG-3′) (Jorgensen et al., 2012). Abundances of the methyl Coenzyme M reductase subunit A (*mcrA*) gene in these river sediments were estimated by qPCR. The DNA fragments encoding the *mcrA* gene were amplified using PCR with the universal primers mlas-mod-F (5′-GGYGGTGTMGGDTTCACMCARTA-3′) and mcrA-rev-R (5′-CGTTCATBGCGTAGTTVGGRTAGT-3′) (Angel et al., 2012). The program used in this procedure involved heating the sample at a temperature of 98°C for 3 min, followed by 40 heating cycles for 15 s at 98°C, 20 s at 58°C, and 30 s of extension at 72°C. Finally, a final elongation step was performed at 72°C for 10 min. The PCR products were purified using a gel extraction kit (DP219-02, TIANGEN, China). The purified PCR products (10 μL) mixed with 2 × Taq PCR Mix (B639295, Sangon Biotech, China) in equal amounts at 72°C for 30 min were added to the end of the sequence. Then, 4 μL of the sequence solution was mixed with 5 μL of solution I and 1 μL pMD18-T vector provided by the pMD 18-T Vector Cloning Kit (6601, TAKARA, Japan). In total, 10 μL of the mixture was incubated at 16°C for 30 min. Then, a vial of DH5α competent cells (CD201-02, Trans, China) was thawed on ice. A 10-μL reaction mixture was added to 50 μL of DH5α competent cells and incubated on ice for 30 min after gentle mixing. The sample was heat shocked for 45 s at 42°C and then chilled on ice for 2 min. Then, 1 mL of Lysogeny broth (LB, tryptone 10 g/L, yeast extract 5 g/L, NaCl 10 g/L) medium was added from the competent cells kit to the transfected cells, followed by sample incubation at 37°C with shaking speed of 200 × RPM for 1 h. Then, the samples were centrifuged at 1,500 × g for 5 min, and 800 μL of the supernatant was removed from the tube. The cells were resuspended in the rest of the medium and then spread onto the solid LB medium with 100 μg/mL of ampicillin. The plate was incubated at 37°C overnight in an inverted position. Single colonies were selected for PCR with primers. The plasmid was extracted by DiaSpin plasmid DNA mini kit (B110091, Sangon Biotech, China). The concentration of the plasmid extract was measured using a NanoDrop 2000 spectrometer (NanoDrop 2000/2000C, Thermo Fisher Scientific, USA).

### 2.4 Gene quantification performed by qPCR

This template used in qPCR for 16S rRNA and *mcrA* gene quantification is the incubation samples. Each reaction contained 20 μL 2 × PowerUp™ SYBR Master Mix (A25742, Applied Biosystems, USA), 2 μL of template DNA, and 1 μL of each forward and reverse primer. The standard curve consisted of a diluted known amount of purified PCR product obtained from plasmid DNA using the bacterial 16S rRNA gene-specific primers bac341f/519r between 10^3^ and 10^9^ copies/μL. The amplification efficiency was between 90–110%, and the R^2^ of the standard curve was above 0.90. The thermal cycle program was for 2 min at 50°C and 3 min at 98°C, followed by 40 cycles for 15 s at 98°C, 30 s at 55°C, and 30 s at 72°C. The standard curve consisted of a known amount of diluted purified PCR product obtained from plasmid DNA using the *mcrA* gene-specific primers mlas-mod-F/mcrA-rev-R between 10^2^ and 10^7^ copies/μL. The thermal cycling conditions were followed by heating at 50°C for 2 min, 98°C for 3 min, followed by 40 cycles of heating at 98°C for 30 s, 58°C for 40 s, and 72°C for 30 s. Three replicates were performed for each sample, and the statistical analysis was performed using Student's *t*-test.

### 2.5 Environmental parameter determination

The soil was first freeze-dried, ground, and sieved. After removing the inorganic carbon from the soil using HCl and re-drying the samples, the organic carbon content (total carbon and total organic carbon) was measured using an element analyzer (Vario EL *III*, Elementar, Germany). The procedure for measuring the total nitrogen content was similar but lacked the reaction with acid. Based on repeated determinations, the detection limits for carbon and nitrogen were 8 μg, with a precision better than 6%. The soil samples used in the above parameters were stored at −20°C. The CH_4_ and CO concentrations in the 20 mL serum bottle headspace were measured on a gas chromatograph with a flame ionization detector (GC-FID, GC-14B, Shimadzu, Japan) (Treat et al., 2014). For gas estimation, each gas sample (1 mL) was manually injected using an airtight syringe (81356, Hamilton, Switzerland). The CH_4_ and CO concentrations in the sample were calculated by external calibration using a certified gas mixture with 50% CH_4_ and 50% CO. The CH_4_ and CO gas peaks were identified based on the retention time of standard CH_4_ and CO gases. The response factor obtained was used to calculate the CH_4_ and CO percentages in the incubation samples.

## 3 Results

### 3.1 Bacterial 16S rRNA gene copy numbers and diversity composition

**Figure 2:**
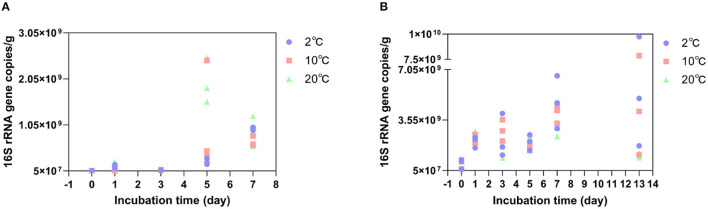
Bacterial 16S rRNA gene copy numbers change at different temperatures. **(A)** is the GR site, and **(B)** is the GR-0 site.

The copy numbers of the bacterial 16S rRNA gene fragments in each DNA fraction were quantified by qPCR. The amplification efficiency ranged between 90 and 110%. At the GR site where the soil was incubated at 2°C (*in situ* temperature. Figure 2A), the average bacterial 16S rRNA gene copy numbers increased from 5.06 × 10^7^ copies/g to 9.73 × 10^8^ copies/g on Day 7. It increased from 5.06 × 10^7^ copies/g to 1.17 × 10^9^ copies/g on the 5^th^ day at 10°C and from 5.06 × 10^7^ copies/g to 1.97 × 10^9^ copies/g on Day 5 at 20°C. The bacterial 16S rRNA gene copy numbers peaked on the 5^th^ day at 10°C and 20°C, compared to those incubated at 2°C. At the GR-0 site where the soil was incubated at 2°C (Figure 2B), the average bacterial 16S *rRNA* gene copy numbers increased from 5.37 × 10^8^ copies/g to 4.78 × 10^9^ copies/g on Day 7. It increased from 5.37 × 10^8^ copies/g to 4.01 × 10^9^ copies/g on Day 7 at 10°C and to 2.93 × 10^9^ copies/g on Day 7 at 20°C. However, the average bacterial 16S rRNA gene copy numbers increased at 2°C, 10°C, and 20°C with an increase in incubation time. In summary, the shift in bacterial 16S rRNA gene copy numbers of 39 GR and 48 GR-0 soil samples had no obvious difference with an increase in incubation temperatures: As the value of the Student's *t-*test is higher than 0.05, there was no significant difference.

**Figure 3:**
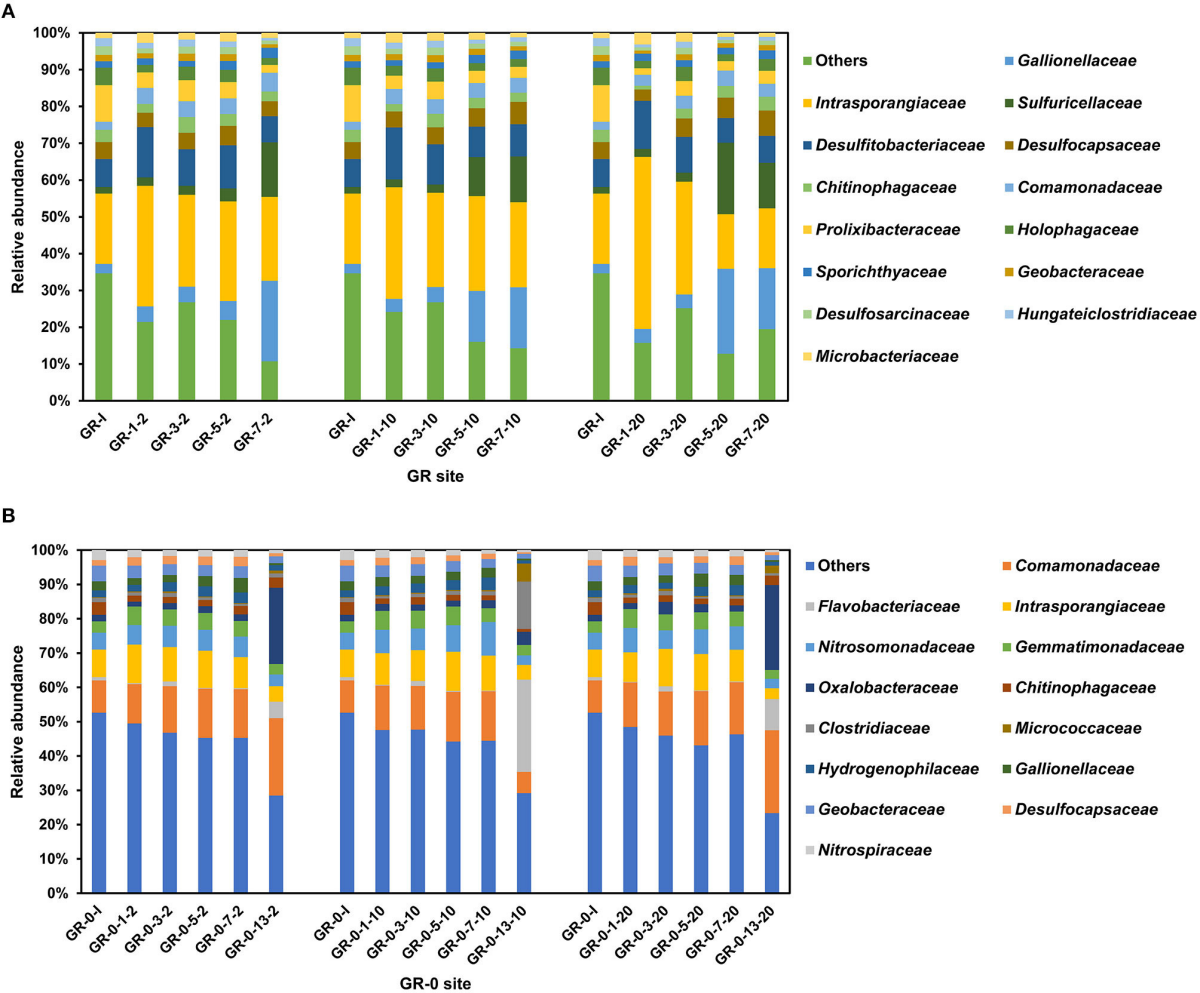
Relative abundance of bacterial community based on 16S rRNA gene at the family level. **(A)** is the GR site, and **(B)** is the GR-0 site. The first number is incubation day, and the third is incubation temperature. “I” means initial samples.

Bacterial community composition was determined for each of the 87 incubation soil samples based on the 16S rRNA gene. The results show that the proportion of high-quality sequences is between 94.56 and 98.86%. The amplicon sequence variants (ASVs) belonging to different phyla have been found in 87 incubation samples at two different sites, totaling up to 1,011,481. The taxa at the GR site were dominated by six bacterial phyla: Actinobacteria (22–61%), Proteobacteria (11–50%), Firmicutes (8–18%), Bacteroidota (3–17%), Desulfobacterota (5–10%), and Acidobacteriota (2–6%) followed by Gemmatimonadota and Chloroflexi (Supplementary Figure 1A). Actinobacteria, one of the most widely distributed phyla among soil bacteria, are well known for their ability to degrade plant residues (cellulose) (Bao et al., 2019, 2021). However, at the GR-0 site, the dominant phyla were Proteobacteria (27–65%), Actinobacteriota (8–21%), Bacteroidota (4–15%), Desulfobacterota (2–7%), Gemmatimonadota (2–5%), and Firmicutes (1–20%), followed by Acidobacteriota and Nitrospirota (Supplementary Figure 1B). Those bacterial species have been reported as dominant groups in the other Svalbard regions (Son and Lee, 2022; Tian et al., 2022). At the family level (Figure 3), the bacterial community composition of the two sites did not show any significant difference with the increase in temperature and incubation time. The Shannon diversity metrics were invariable between samples ranging from 5 to 8 (GR) and 8 to 10 (GR-0). No statistically significant difference was found between the different sample types. Based on the differences at the phylum level, the two sites show differences at the family level. At the GR site, *Intrasporangiaceae, Gallionellaceae, Sulfuricellaceae*, and *Desulfitobacteriaceae* were the dominant groups (Figure 3A). Meanwhile, at the GR-0 site, *Comamonadaceae, Intrasporangiaceae, Nitrosomonadaceae*, and *Gemmatimonadaceae* were the main groups (Figure 3B).

### 3.2 CH_4_ production potential

**Figure 4:**
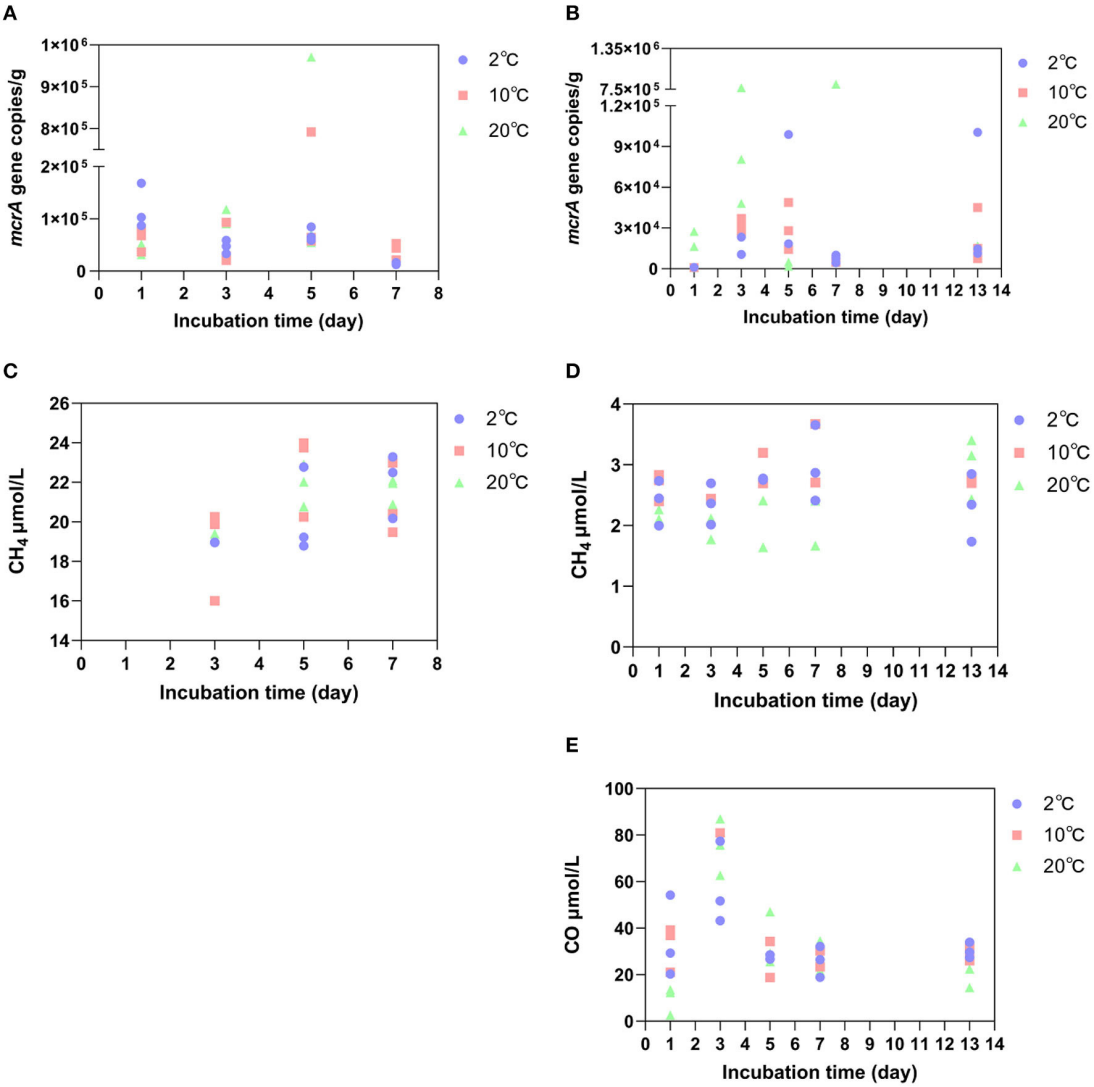
The abundance of the *mcrA* gene at the GR site **(A)** and GR-0 site **(B)**, as well as the concentration of CH_4_ at the GR site **(C)** and GR-0 site **(D)**. The concentration of CO at the GR-0 site **(E)**.

To determine the abundance of methanogenic archaea, we quantified the functional gene *mcrA*, which encodes methylcoenzyme M reductase and is a key enzyme in methanogenesis (Inagaki et al., 2004). Among the incubation samples at the GR site, the average copy number of *mcrA* genes reached the maximum on the 5^th^ day at 10°C and 20°C, reaching 3.1 × 10^5^ copies/g and 3.7 × 10^5^ copies/g, respectively, except for 2°C, where they did not reach the maximum before decreasing. While, at 2°C, the average copy number of *mcrA* genes decreased from 1.2 × 10^5^ copies/g on the first day to 1.4 × 10^4^ copies/g on Day 7 (Figure 4A). However, the concentration of CH_4_ in the soil showed an increasing trend during the incubation process at all three warming temperatures. Under 2°C, the average net increase of CH_4_ for 2 days (from the third- to the fifth-day incubation) was 1.3 μmol/L. Under 10°C, the average net increase of CH_4_ for 2 days was 3.9 μmol/L. At 20°C, the average net increase of CH_4_ was 2.4 μmol/L for 2 days (Figure 4C). Compared to GR site incubation samples, the copy number of *mcrA* genes at the GR-0 site changed irregularly (Figure 4B). In addition, the concentration of CH_4_ at the GR-0 site was an order of magnitude lower than that at the GR site, and there was no substantial variation in CH_4_ concentration (Figure 4D).

### 3.3 Variations in temperature and soil environmental variables

When determining the gas content during the incubation period, we detected not only CH_4_ gas but also carbon monoxide (CO) in incubated samples. However, it only exists at the GR-0 site. At all three different incubation temperatures, the concentration of CO was higher in the early stage of incubation (i.e., the first 3 days) and reached its maximum on the third day (Figure 4E), which was 57.4 μmol/L at 2°C, 90 μmol/L at 10°C, and 75.2 μmol/L at 20°C.

The environmental variables of the three incubation temperatures are shown in Table 1. As shown by the results, the contents of total organic carbon (TOC), total carbon (TC), and total nitrogen (TN) showed no obvious differences in the short-term warming field experiments of the GR and GR-0 soil samples. The content of TOC is between 0.8 and 1.4%, the content of TC is between 1.0 and 1.1%, and the content of TN is approximately 0.1%. In addition, the C/N ratio (TOC/TN) among incubation soil samples was <15, ranging from 7.7 to 14.2. At the GR site, the C/N ratio gradually increased to 14.2 under 20°C conditions. However, at the GR-0 site, the C/N ratio increased in the first 7 days at all three incubation temperatures and then decreased from Day 7 to Day 13. The mental test showed that soil CO (0.2 <Mental's r <0.4, Mental's *p* <0.05), CH_4_ (0.2 <Mental's r <0.4, Mental's *p* <0.01), and TN (0.2 <Mental's r <0.4, Mental's *p* < 0.05) were the major factors affecting soil microbial composition (Supplementary Figure 2).

**Table 1:** The average environmental value of the soil incubated at different temperatures.

		**Incubation temperature**											
		**2**°**C**				**10**°**C**				**20**°**C**			
**Site ID**	**Incubation time (days)**	**TOC (%)**	**TC (%)**	**TN (%)**	**C/N ratio**	**TOC (%)**	**TC (%)**	**TN (%)**	**C/N ratio**	**TOC (%)**	**TC (%)**	**TN (%)**	**C/N ratio**
GR	0	-	-	-	-	-	-	-	-	-	-	-	-
	1	1.1	1.0	0.1	10.5	0.9	1.0	0.1	8.7	0.8	1.0	0.1	7.9
	3	1.1	1.0	0.1	10.5	1.1	1.0	0.1	11.4	0.9	1.0	0.1	9.3
	5	1.2	1.0	0.1	11.3	1.1	1.0	0.1	10.9	1.2	1.0	0.1	11.3
	7	0.8	1.0	0.1	7.8	0.8	1.0	0.1	7.8	1.4	1.0	0.1	14.2
GR-0	0	0.8	1.0	0.1	8.8	0.8	1.0	0.1	8.8	0.8	1.0	0.1	8.8
	1	-	-	-	-	-	-	-	-	-	-	-	-
	3	-	-	-	-	-	-	-	-	-	-	-	-
	5	0.8	1.0	0.1	8.4	0.9	1.0	0.1	10.0	0.8	1.0	0.1	9.0
	7	1.3	1.0	0.1	14.0	1.0	1.0	0.1	11.0	1.1	1.0	0.1	11.2
	13	0.7	1.0	0.1	7.9	0.8	1.1	0.1	8.8	1.0	1.1	0.1	10.4

“-” means no data.

## 4 Discussion

### 4.1 Effects of short-term warming on microbial composition in Arctic soils

There were no changes in microbial composition during short-term soil warming, measured in days, under climate change (Figure 3). Previously, using 454 pyrosequencing of 16S rRNA genes, it was found that warming (+0.5 to 2°C) with open-top chambers for 3 years had altered the soil bacterial communities at two locations in the maritime Antarctic and one in the cool southern temperate zone, with consistent increases observed across all three locations in Alphaproteobacteria-to-Acidobacteria ratios (Yergeau et al., 2012). A study on long-term warming observed that slow-growing bacteria (K-selected), such as Gram-positive Actinobacteria, increased in dominance with warming at Toolik Lake in Alaska. This suggests that the increased dominance of these recalcitrant C-recyclers suggests a reduction in the availability of labile substrates with warming (Deslippe et al., 2012). However, a meta-analysis of field studies indicates that day, diurnal, and night warming had no effect on overall bacterial abundance, and no significant between-group heterogeneity was found for various measurement methods (Chen et al., 2015). Our results showed that no matter how the temperature changed, the microbial biomass (16S rRNA gene copy number) increased during the first 7- and 13-day incubation at 2°C, 10°C, and 20°C (Figure 2). Increases in microbial biomass and activity may have happened in a short-term climate change. However, the limitation of mineral nutrients such as nitrogen may constrain this response in the long term. Such mineral limitation will affect the dominance of oligotrophic and copiotrophic microorganisms in a given ecosystem, which in turn may influence GHG fluxes (Singh et al., 2010; Romero-Olivares et al., 2017).

We clustered and analyzed the bacterial community composition through complete linkage, using the largest numerical distance between two datasets as the distance between two groups for pairwise comparison to obtain data similarity between groups. The closer the distance, the shorter the branch distance of the cluster. Our results show samples of the same incubation time clustered on a branch (Supplementary Figure 3), and the microbial composition of the two sites (GR and GR-0) differed (Figure 3). However, the bacterial community composition showed no difference during warming incubation for 7 and 13 days, with no changes in the dominant species or the relative abundance of each community (Figure 3). The influence of incubation time and location along the river on the microbial community was greater than that of incubation temperatures. Since microorganisms adapt to grow in a specific temperature range, when the temperature fluctuates within its growth range, the microbial composition will not change obviously for the short term (Rijkers et al., 2022). However, due to a series of influences, such as vegetation type, soil water content, and soil depth, the microbial composition at different sampling sites showed obvious differences after long-term domestication (Son and Lee, 2022). These findings also agree with results from other Arctic tundra climate change experiments showing a strong response of soil microbial communities to vegetation types and spatial scale (Campbell et al., 2010; Malard et al., 2021).

Meanwhile, at the upper stream of the glacial meltwater river, Actinobacteriota is the dominant group. The average number of CAZyme enzyme genes encoded in the genome of Actinomycetes is higher, and plant-derived organic matter can be used in soils with declining soil fertility (Bao et al., 2021). This result is consistent with the utilization of terrigenous organic matter by the bacteria in the upper stream of the river. The dominant group in the glacial meltwater river's estuary is Gammaproteobacteria, belonging to Proteobacteria. The class Gammaproteobacteria is known as one of the denitrifier groups, and many species of this class are cold-adapted. It thus might be an important group to determine the capacity of Arctic rivers to remove excess nitrogen (Franco et al., 2017; Uchida et al., 2018; Qian-Qian et al., 2021). More importantly, long-term warming rather than short-term induced changes in the composition of soil microbial communities can cause sustained changes in microbial activity, resulting in soil carbon emissions.

### 4.2 Effects of short-term warming on CH_4_ release from Arctic soils

CH_4_ emissions caused by warming on microbial metabolisms may be a long-term process measured by months or years rather than several days. A meta-analysis of field studies shows that soil microbial respiration causes carbon losses by 1–5 years of warming incubation. In comparison, it suggests that soil carbon losses decrease after long-term warming, especially after 10 years (Romero-Olivares et al., 2017). In addition, studies have documented changes in the CH_4_ concentration of the Lagoon Pingo surface areas from April 2016 to October 2017, with the CH_4_ concentration of 906.3 μmol/L in April 2016 and 601.9 μmol/L in March 2017. However, from 6 August 2017 to 24 August 2017, CH_4_ concentrations were 338.1 μmol/L and 383.1 μmol/L, respectively. It can be concluded that the CH_4_ emissions do not change much in the short term (Hodson et al., 2019). It is consistent with our results that CH_4_ concentration at two different sites did not change with the increase of temperature (2°C, 10°C, and 20°C) and time (7 and 13 days) during incubation (Figures 4C, D). Moreover, Alaskan tundra soils at a depth of 45–55 cm were subjected to experimental *in situ* warming by nearly 1.1°C above ambient temperature, which corresponded with a 3-fold increase in the abundance of a single archaeal clade of the *Methanosarcinales* order and accompanied a comprehensive increase in the relative abundances of methanogenesis genes after 2-month incubation (Johnston et al., 2019). A whole-soil-profile 3-year warming experiment suggests that short-term warming does not alter microbial carbon use efficiency in either surface or deep soils (Zhang et al., 2023a). In our research, methanogenic gene abundance (*mcrA* gene copy numbers) in our short-term incubation did not increase with the anoxic warming experiment (Figures 4A, B). Nevertheless, a large amount of CO was detected at the GR-0 site (Figure 4E), which may be a product of microbial fermentation or incomplete oxidation under oxygen restriction (Terry et al., 2004; Diender et al., 2015). CO, as a chemically active gas, although its direct greenhouse effect is negligible, directly oxidizes the hydroxyl radicals in the atmosphere, becoming the main sink for hydroxyl radicals and hence being beneficial for the accumulation of CH_4_ in the atmosphere (Borsdorff et al., 2019).

The effect of time on warming-induced carbon losses is described as follows: the results of our 13-day warming field experiment showed that warming did not affect the CH_4_ concentration and methanogenic gene abundance. In the first few weeks of temperature rise, microbial metabolisms in response to environmental changes take time to accumulate (Voolstra and Ziegler, 2020), and the amount of change in its products must also accumulate. It concluded that warming-induced changes in the microbial community in the Arctic soils over a few weeks to months would amplify the instantaneous increase in the rates of CO_2_ production and thus enhance carbon losses (Hartley et al., 2008). However, declines in the response of microbial respiration to warming in long-term experiments (>5 years) suggest that microbial activity acclimates to temperature, greatly reducing the potential for enhanced carbon losses (Hartley et al., 2008). Therefore, we suggested that CH_4_ emissions in the process of soil warming have no increase in the short term, and with the increase in warming time, emissions will gradually increase in the long term. On the other hand, when microorganisms adapt to warming, CH_4_ emissions will gradually decrease.

### 4.3 The factors affecting CH_4_ production in the Arctic

Many factors, such as oxygen, moisture, vegetation type, seasonal change, and temperature, affect the product of CH_4_ in the Arctic. Finally, CH_4_ production is closely related to microorganisms in the Arctic soil, one of the most important areas of CH_4_ emissions. Some studies suggest that warming surface soil may increase CO_2_ emissions, while CH_4_ production is more prevalent in deeper soils (Knoblauch et al., 2021; Galera et al., 2023). As mentioned above, when considering net emissions of CH_4_ in soil with the anaerobic methanogenic archaea as the source and the trophic methanogenic oxidizing bacteria as the sink, the net CH_4_ production value in the soil only occurs when the two cancel out. The study indicated that CH_4_ flux was more strongly influenced by long-term gradients in soil moisture and vegetation than plant biomass, species composition, or nutrient availability (Torn and Chapin, 1993). This view is consistent with our experimental results: the difference in CH_4_ concentration between the GR and GR-0 sites is an order of magnitude (Figures 4C, D), which may be caused by soil moisture. One of the primary reasons for the microhabitat differences within the soil is the soil water content, where methanotrophs require oxygen and methanogens are anaerobic (Freitag et al., 2010; Galera et al., 2023).

**Figure 5:**
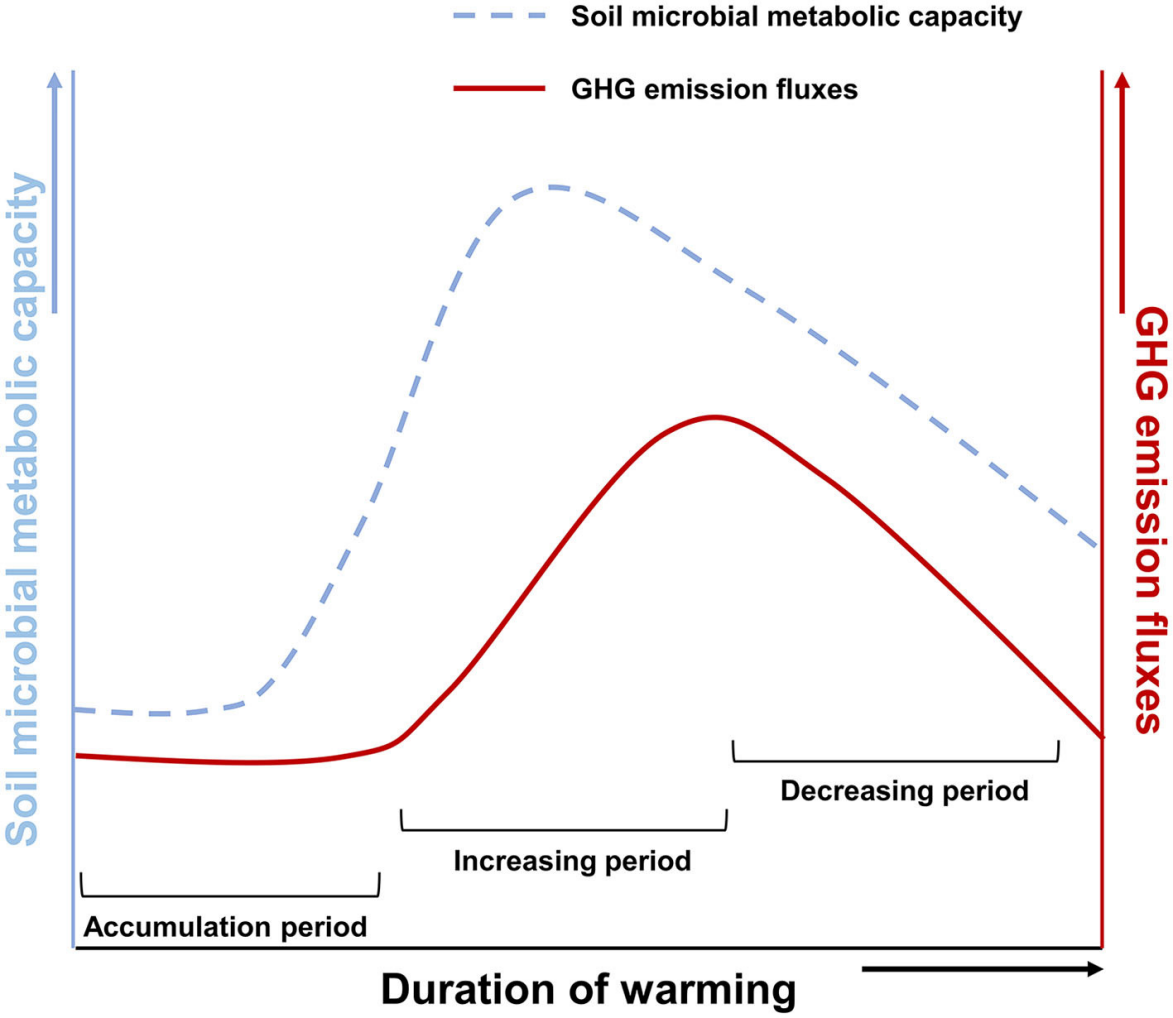
A schematic diagram of warming-induced soil microorganism respiration and GHG emission fluxes. The dotted line indicates microbial metabolic capacity. Solid lines show GHG emission fluxes.

To sum up, regardless of the influence of environmental factors, warming might take time to accumulate to affect Arctic soil microbial respiration, the main metabolic activity in Arctic soil (Nazaries et al., 2013; Tveit et al., 2013; Hamdan and Wickland, 2016; Knoblauch et al., 2018; Galera et al., 2023). It was altering GHG emission fluxes (Figure 5; accumulation period). It takes several months, even years, for GHG produced by microorganisms to be released from the soil into the atmosphere, so there is a lag. Besides, the effect of time on it is not a continuous positive correlation (Figure 5; increasing period). As time passes, this effect shows a trend of increasing and gradually weakening (Figure 5; decreasing period).

## 5 Conclusion and future perspectives

In summary, the warming field experiment was conducted by anaerobic incubating surface soil samples at two sites in the upper reaches and estuaries of the Barentsburg glacial meltwater river for 7 and 13 days. The results showed that the microbial composition at 10°C and 20°C was not different from that at 2°C. There was also no difference in soil microbial methanogenic gene abundance and CH_4_ concentration after incubation. Therefore, we conclude that the acceleration of microbial respiration caused by warming will increase the CH_4_ flux over at least 2 weeks. It is interesting that the GR-0 river bank site released more CO compared to the GR site, which did not emit any CO. The effects of global warming on microbial metabolisms and soil CH_4_ emission fluxes could be studied through longer-term and continuous incubation experiments or observation. The above conclusions provide reference data for assessing CH_4_ emission fluxes in the Arctic region and ideas for future research on the impact of warming on CH_4_ emissions.

## Data availability statement

The datasets presented in this study can be found in online repositories. The names of the repository/repositories and accession number(s) can be found in the article/Supplementary material.

## Author contributions

JL: Conceptualization, Data curation, Formal analysis, Funding acquisition, Investigation, Methodology, Project administration, Resources, Software, Supervision, Validation, Visualization, Writing – original draft, Writing – review & editing. Z-YZ: Conceptualization, Data curation, Formal analysis, Funding acquisition, Investigation, Methodology, Project administration, Resources, Supervision, Validation, Visualization, Writing – review & editing. ZY: Data curation, Formal analysis, Investigation, Methodology, Resources, Writing – review & editing. WL: Methodology, Software, Visualization, Writing – review & editing. YL: Software, Writing – review & editing. YZ: Conceptualization, Funding acquisition, Validation, Writing – review & editing.
