# Supplementary material for: Soil microorganisms and methane emissions in response to short-term warming field incubation in Svalbard
Source: Front Microbiol. 2023 Nov 24;14:1276065. doi: 10.3389/fmicb.2023.1276065 (PMC10704465; doi:10.3389/fmicb.2023.1276065)
Supplement: Supplementary file 1 [file Data_Sheet_1.docx]

Supplementary Material

## Supplementary Figures





**Supplementary Figure 1.** Relative abundance of bacterial community based on 16S rRNA gene at the phyla level. (A) is the GR site, and (B) is the GR-0 site. I: initial samples, the second number is incubation day, and the third is incubation temperature.


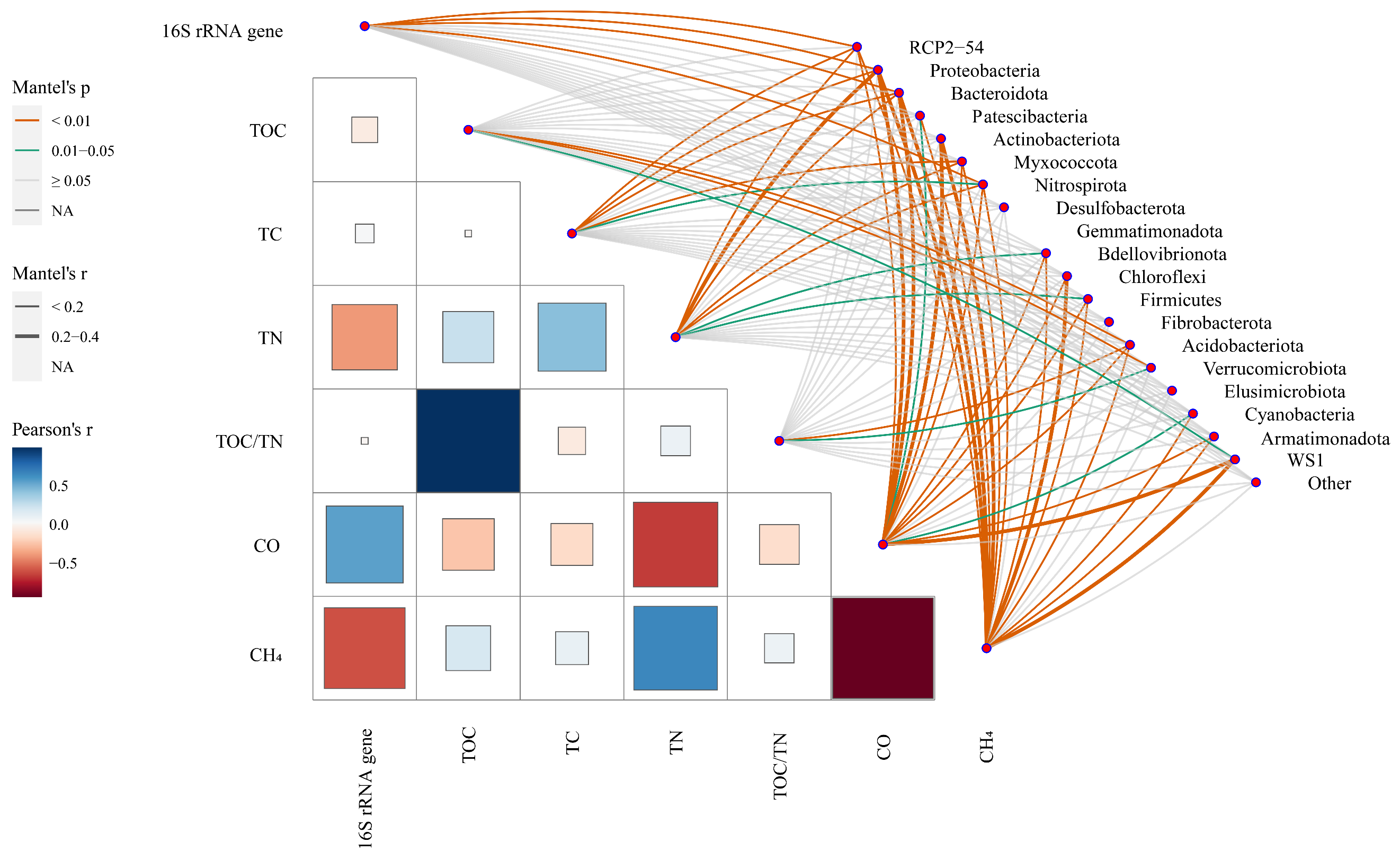


**Supplementary Figure 2.** Mantel test to determine the correlation between soil properties and soil microbial composition. the significance level at 0.05≤ Mental’s p < 0.01, the significance level at 0.2≤ Mental’s r < 0.4. Blue is a strong positive correlation; white is a weak correlation; red is a strong negative correlation.


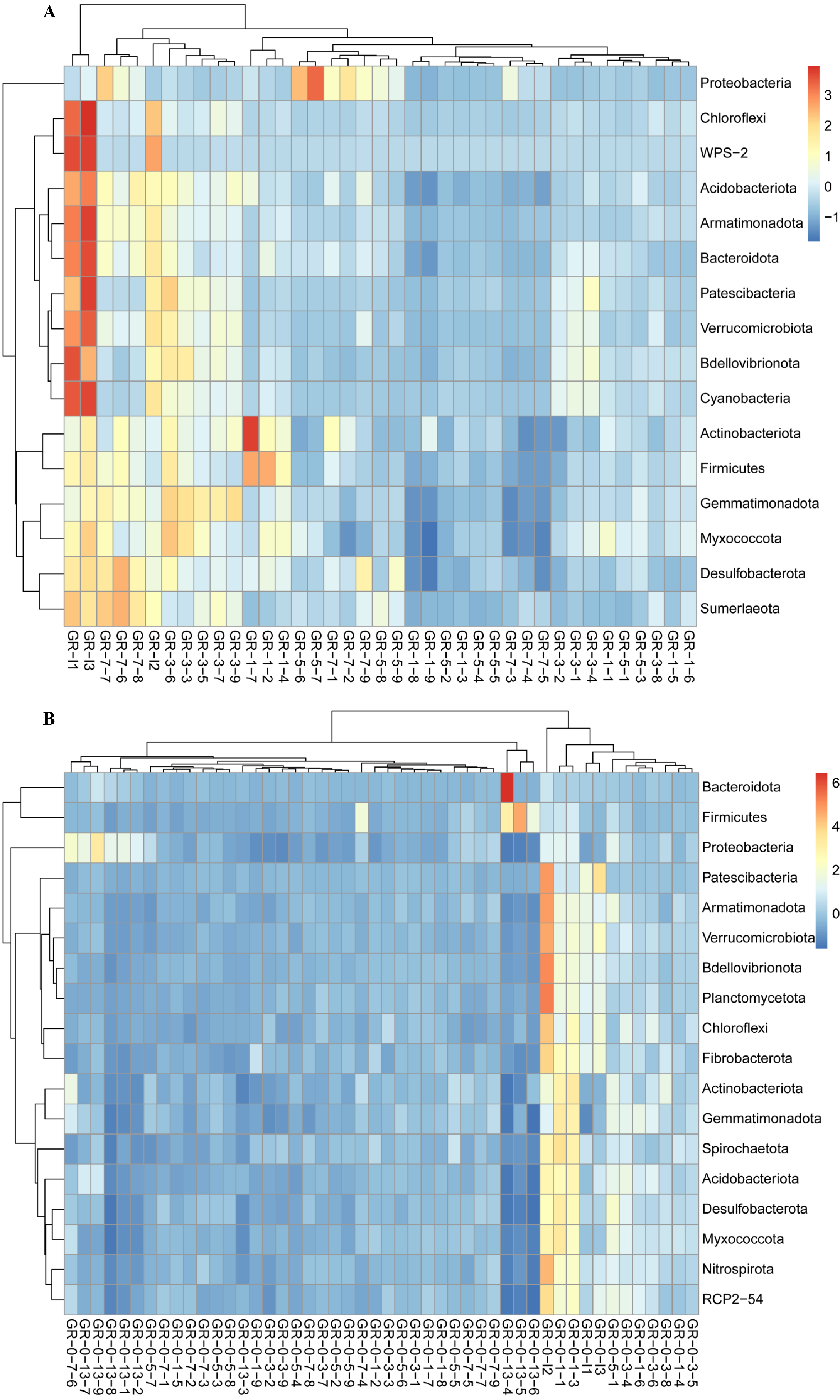


**Supplementary Figure 3.** The heatmap of bacterial phylum with a relative abundance ≥ 0.1% at the GR site **(A)** and the GR-0 site **(B)**. The color represents species abundance. The horizontal coordinate indicates different incubation temperatures and times. I1-3: three initial replicates samples; the first number is incubation time; the second number: 1-3 means three replicates at 2℃; 4-6 means three replicates at 10℃; 7-9 means three replicates at 20℃.

## Data availability statement

All bacterial 16S rRNA gene sequences have been deposited in The National Omics Data Encyclopedia database under the BioProject accession number OEP004357 and OEP004358.
